# Chemical Constituents from the Wild *Atractylodes macrocephala* Koidz and Acetylcholinesterase Inhibitory Activity Evaluation as Well as Molecular Docking Study

**DOI:** 10.3390/molecules26237299

**Published:** 2021-12-01

**Authors:** Qiannan Zhu, Min Lin, Wanying Zhuo, Yunzhi Li

**Affiliations:** 1 School of Pharmacy, Anhui University of Chinese Medicine, Hefei 230031, China; qiannanzhu@aliyun.com (Q.Z.); Linmin_529@163.com (M.L.); zhuowy998@163.com (W.Z.); 2 Department of Medicinal Chemistry, Anhui Academy of Chinese Medicine, Hefei 230012, China

**Keywords:** biatractylenolide II, acetylcholinesterase inhibitory activity, molecular docking

## Abstract

Screening the lead compounds which could interact both with PAS and CAS of acetylcholinesterase (AChE) is an important trend in finding innovative drugs for Alzheimer’s disease (AD). In this paper, four sesquiterpenes, i.e., atractylenolide III (**1**), atractylenolide IV (**2**), 3-acetyl-atractylon (**3**) and β-eudesmol (**4**), were obtained from the wild *Atractylode macrocephala* grown in Qimen for the first time. Their structures were elucidated mainly by NMR spectroscopy. To screen the potential dual site inhibitors of AChE, the compounds **1**, **2**, **3,** as well as a novel and rare bisesquiterpenoid lactone, biatractylenolide II (**5**), which was also obtained from the tilted plant in our previous investigation, were evaluated their AChE inhibitory activities by using Ellman’s colorimetric method. The results showed that biatractylenolide II displayed moderate inhibitory activity (IC_50_ = 19.61 ± 1.11 μg/mL) on AChE. A further molecular docking study revealed that biatractylenolide II can interact with both the peripheral anionic site (PAS) and the catalytic active site (CAS) of AChE. These data suggest that biatractylenolide II can be considered a new lead compound to research and develop more potential dual site inhibitors of AChE.

## 1. Introduction

Currently, Alzheimer’s disease (AD) is one of the most difficult progressive neurodegenerative disorders to treat [1]. Among various pathogenesis hypotheses of AD, cholinergic hypothesis and amyloid-β aggregation are two widely accepted theories [2,3]. According to the cholinergic hypothesis, restoring the level of acetylcholine by using reversible inhibitors to inhibit cholinesterase is a possible approach to treat AD. Research has shown that there is a deep, narrow gorge in its crystal structure of AChE. The catalytic active site (CAS) of AChE is located at the bottom of the gorge, while the peripheral anionic site (PAS) is at the entrance [4]. A previous investigation demonstrated that AChE is able to accelerate amyloid-β aggregation, while such an effect is affected when drugs block PAS of the enzyme [5,6]. Therefore, it is an important trend to screen the lead compounds which could interact both with PAS and CAS of the enzyme in AD drug R&D [7]. 

*Atractylodes macrocephala Koidz* (Asteraceae) is mainly distributed in East Asia. In China, the rhizome of *A. macrocephala* has been used as food and traditional Chinese medicine for thousands of years [8]. Its main biological functions in Chinese medicine are to enhance immunity, strengthen the spleen, eliminate dampness, promote diuresis, stop sweat, and prevent miscarriage. Now, most of the *A. macrocephala* sold in the Chinese herbal medicine market are almost all cultivated species that originate from the Yuqian district (Yu-Zhu), Zhejiang Province, China. In fact, there is a wild *A. macrocephala* (Qi-Zhu) grown in Qimen district, Anhui Province which is also a famous regional drug. In contrast to Yu-Zhu, Qi-Zhu only grows in the wild at 800 metres above sea level. There are different efficiencies between them. For example, Qi-Zhu can treat jaundice and ascites, whereas Yu-Zhu does not have these functions [9]. In addition, they have different plant morphologies [9]. Based on these differences, we speculate that Qi-Zhu should have different secondary metabolites than Yu-Zhu, which was confirmed by our previous study [10]. The previous phytochemical investigation found a series of lactone compounds displaying antitumour, anti-inflammatory, gastrointestinal adjustment and absorption-promoting activities [11]. However, to date, little is known about the chemical constituents of Qi-Zhu. Our previous investigation only isolated three compounds, including a novel bisesquiterpenoid lactone, biatractylenolide II, together with two known compounds, atractylenolide II and taraxeryl acetate, from its rhizome [12]. In this study, we carried out a further phytochemical investigation into this herb and found four sesquiterpenes, i.e., atractylenolide III (**1**), atractylenolide IV (**2**), 3-acetyl-atractylon (**3**) and β-eudesmol (**4**), for the first time. Here, we report their isolation. In addition, the previous investigation showed that a bisesquiterpenoid lactone, biatractylenolide, which was obtained from cultivated *A. macrocephala*, exhibited acetylcholinesterase (AChE) inhibitory activity [13]. Biatractylenolide II is also a bisesquiterpenoid lactone isolated by us from Qi-Zhu [12]. As an analogue of biatractylenolide, we deduced that biaraliactylenolide II may have the same activity. To clarify this hypothesis, we evaluated the effect of biatractylenolide II on AChE in this paper. At the same time, the other atractylenolide lactones obtained from this herb were also assayed for AChE inhibitory activity. Furthermore, for screening the potential dual site inhibitors of AChE, a molecular docking was used to study the interaction between biatractylenolide II and AChE in this paper.

## 2. Results and Discussion

### 2.1. Chemistry 

**Figure 1:**
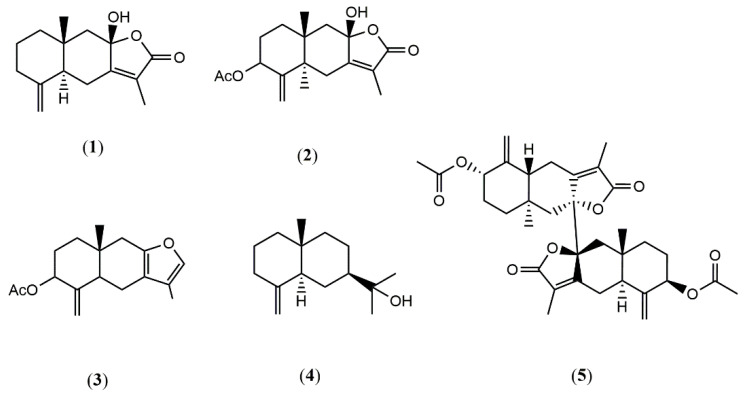
The chemical structure of compounds **1–5** from the wild *A. macrocephala*.

According to the procedure described in Materials and Methods, four sesquiterpenes (**1–4**), i.e., atractylenolide III (**1**), atractylenolide IV (**2**), 3-acetyl-atractylon (**3**) and β-eudesmol (**4**), and a bisesquiterpene, biatractylenolide II (**5**), were obtained from the rhizome of *A. macrocephala*. Their chemical structures were mainly elucidated by NMR and MS spectra and are shown in Figure 1.

Atractylenolide III (**1**). C_15_H_20_O_3_. White needle crystal (Petroleum ether -AcOEt), mp 183–185 °C. ESI-MS: m/z 249 [M + H]^+^. ^1^H and ^13^C NMR data and MS data were consistent with the previously published data in the literature [14,15], and **1** was identified as atractylenolide III.

Atractylenolide IV (**2**). C_17_H_22_O_5_. mp 210–212 °C. ESI-MS: m/z 289.1 [M + H]^+^. ^1^H and ^13^C NMR data and MS data were consistent with the previously published data in the literature [16], and **2** was identified as atractylenolide IV.

3-Acetyl-atractylon (**3**). C_17_H_22_O_3_. mp 93–95 °C. ESI-MS: m/z 275 [M + H]^+^. ^1^H and ^13^C NMR data and MS data were consistent with the previously published data in the literature [17], and **3** was identified as 3-acetyl-atractylon.

β-Eudesmol (**4**). C_15_H_26_O. ^1^H and ^13^C NMR data and MS data were consistent with the previously published data in the literature [18], and **4** was identified as β-eudesmol.

Biatractylenolide II (**5**). C_34_H_42_O_8_. ^1^H and ^13^C NMR data and MS data have been reported in the literature [12]. 

Biatractylenolide II is a kind of rare bisesquiterpenoid lactone found in nature. Before this, only two chemical constituents, biatractylenolide [19] and biepiasterolid [20], have been identified with similar structural features obtained from the genus *Atractylodes*. Additionally, bisesquiterpenoids are also rarely found in other genera, such as *Petasites japonicas* [21]. Recently, the bioactivity of biatractylenolide isolated from *A. macrocephala* was investigated and it was suggested that it could improve the studying memory of an Alzheimer’s disease (AD) rats model induced by Aβ1–40 and reduced cholinesterase activity [22], and had a neuroprotective effect against memory impairment induced by glutamate [23]. As an analog of biatractylenolide, it is unclear whether biatractylenolide II exhibits the neuroprotective effect. Based on the relationship between AD and the deficiency of AChE, we evaluated the AChE inhibition bioactivity of biatractylenolide II. The other series of atractylenolide obtained from the titled herb were also evaluated for AChE inhibition activities in this paper for the first time. In addition, molecular docking was further used to study the interaction between biatractylenolide II and AChE.

### 2.2. Biological Activity and Docking Studies

The modified Ellman method was used to evaluate the inhibitory activities of biatractylenolide II against AChE. The results showed that biatractylenolide II had a moderate inhibition activity on AChE with IC_50_ values of 19.61 ± 1.11 μg/mL. In addition, another atractylenolide, atractylenolide III, also showed AChE inhibition activity (IC_50_ values was 61.26 ± 3.01 μg/mL), whereas the other atractylenolide did not display efficiency on AChE. According to the above results, analyzing the structures of the above four compounds (**1**, **2**, **3**, **5**), we deduced that the acetyl group in compounds **2** and **3** may be a functional group which weakens their AChE inhibitory activities.

**Figure 2:**
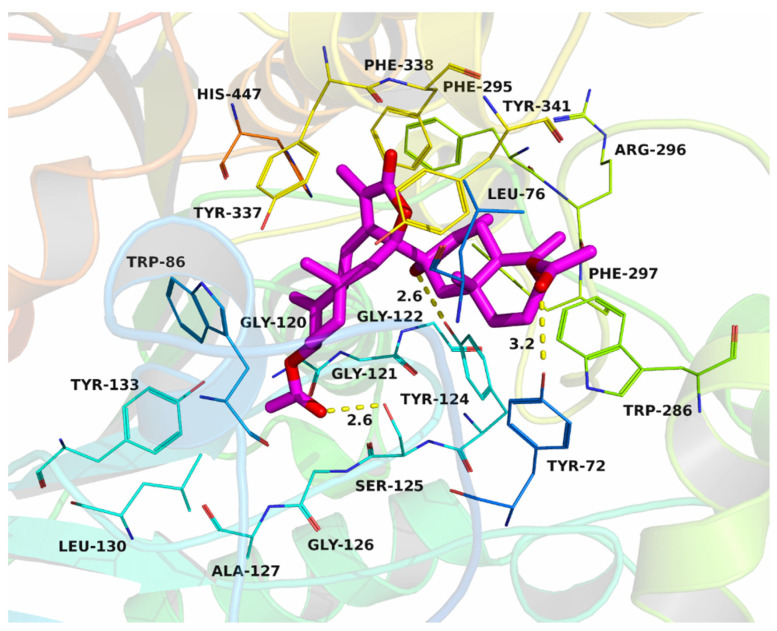
Biatractylenolide II was docked into the binding pocket of AChE.

Based on the results of the inhibition activity of biatractylenolide II on AChE, the interactions between biatractylenolide II and AChE were investigated by molecular docking. The theoretical binding mode of biatractylenolide II in the binding site of the AChE is illustrated in Figure 2. The evaluated binding energy between biatractylenolide II and AChE was −8.2 kcal·mol^−1^. Biatractylenolide II adopted a compact conformation to bind inside of the pocket of AChE. One of the monomers of biatractylenolide II was located at the PAS site, surrounded by the residues Leu-76, Phe-297 and Trp-286, forming a strong hydrophobic binding. The other monomers of biatractylenolide II were positioned in the CAS site that consisted of Trp-86, Phe-295 and Phe-338, forming a stable hydrophobic binding. Detailed analysis showed that one hydrogen bond was observed between the biatractylenolide II and residues Tyr-72, Tyr-124 and Ser-125, with the bond length of 2.6 Å, respectively. All these interactions helped biatractylenolide II to anchor in the two binding sites of AChE.

Although the AChE inhibitory activities of biatractylenolide II are not as good as AChE inhibitors used in clinical settings, such as donepezil, the above molecular simulations suggested that biatractylenolide II could interact with dual sites (CAS and PAS) of AChE. Therefore, biatractylenolide II may have the ability to hinder amyloid-β aggregation, not just as an AChE inhibitor. Therefore, as a lead compound, biatractylenolide II is worthy of further study. Of course, there is still a long way to go to obtain an efficient anti-Alzheimer’s molecule.

In neuroactive drug design, a major problem to overcome is the ability of the compound to cross the blood–brain barrier (BBB). The log *p*-value is a measure of the lipophilicity, which is an important physicochemical property to predict the ability of a drug for the treatment of AD to pass the BBB. Generally, the log *p*-value with the optimum central nervous system penetration is around 2 ± 0.7. In this paper, ChemDraw Ultra 2010 was used to calculate the lipophilicity of biatractylenolide II, and we obtained log *p*-values of 3.47, which suggested that biatractylenolide II was sufficiently lipophilic to cross the blood–brain barrier.

## 3. Materials and Methods

### 3.1. Chemistry

All the chemical solvents used were high-grade commercial products and they were purchased from Sinopharm Group Co. Ltd. (Shanghai, China). Silica gel was purchased from Qingdao Ocean Chemical Industry Co., China. Sephadex LH-20 was obtained from Amersham Biosciences (Uppsala, Sweden). The NMR spectra were recorded on a Bruker AV 400 NMR instrument (Karlsruhe, Germany) (400 MHz for ^1^H NMR, and 100 MHz for ^13^C NMR).

The wild *A. macrocephala* was collected in January 2014 from Xin’an, Qimen district, China, and identified by Prof. Shou-Jin Liu, Department of Crude drug, Anhui University of Chinese Medicine. A voucher specimen (No. lyz 004) was deposited at School of Pharmacy, Anhui University of Chinese Medicine.

The rhizome of *A. macrocephala* (800 g) was air-dried and powdered, extracted three times with 95% EtOH (5 L, 2 h each), then we evaporated the solvent in vacuo to yield 89 g of crude extract. The crude extract was suspended in water, partitioned with AcOEt (3 × 1 L) and n-BuOH (3 × 1 L), successfully, then we removed the solvents by a rotary evaporator at 40 °C, to yield a 35 g AcOEt fraction and a 17 g n-BuOH fraction, respectively. Next, the AcOEt fraction was isolated by a silica gel column using a gradient mixture of petroleum ether-AcOEt (100:0–0:100) to yield six fractions (Y1–Y6). Fr. Y2 was further isolated by silica gel chromatography (petroleum ether-acetone, 15:1), purified by LH-20 chromatography (CHCl_3_-MeOH, 1:1), to give compounds **1** (11 mg), **2** (5 mg), **3** (2 mg) and **4** (19 mg). Atractylenolide II (**5**) was obtained from Fraction Y9 with repeated column chromatography as described in the previous paper [12]. 

### 3.2. Biological Activity

AChE (Type VI-S, from electric eel) was purchased from Yuanye Bio-Technology (Shanghai, China). 5,50-Dithiobis (2-nitrobenzoic acid) (DTNB, Ellman’s reagent) acetylthiocholine iodide (AChI) were obtained from Fluka. Buffer compounds (potassium dihydrogen phosphate, potassium hydroxide) and sodium hydrogen carbonate were purchased from Sigma-Aldrich (Steinheim, Germany). Spectrophotometric measurements were performed on a Molecular Devices SpectraMax i3.

### 3.3. Acetylcholinesterase Activity Assay

A modified colorimetric method of Ellman was used to evaluate the inhibitory effects of the compounds on AChE [24]. Firstly, all solutions were adjusted to 20 °C. Then, both enzyme solution (100 μL) and inhibitor solution (100 μL) were added into a cuvette containing the phosphate buffer (3.0 mL, 0.1 M; pH 8.0). After 5 min incubation, we added required aliquots of the DTNB solution (100 μL) and AChI. Then, rapid and immediate mixing was conducted and we measured the absorption at 405 nm by a Multi-Mode microplate reader. An identical solution of the enzyme without the inhibitor was used as a reference, which was processed following the same protocol. The blank reading contained 3.0 mL buffer, 200 μL water, 100 μL DTNB and 20 μL substrate. The samples were assayed immediately after preparation. The enzyme activities were determined in the presence of at least five different concentrations of a compound. Each concentration was investigated in triplicate.

### 3.4. Molecular Docking

A molecular docking study was performed to investigate the binding mode of the biatractylenolide II to Mus musculus acetylcholinesterase (AChE) using Autodock vina 1.1.2 [25]. The three-dimensional (3D) coordinate of the AChE (PDB ID: 5FUM) was downloaded from Protein Data Bank (http://www.rcsb.org/pdb/home/home.do (accessed on 26 March 2017)). The 3D structure of biatractylenolide II was drawn by ChemBioDraw Ultra 12.0 and ChemBio3D Ultra 12.0 softwares. The AutoDockTools 1.5.6 package [26,27] was employed to generate the docking input files. The search grid of AChE was identified as center_x: 33.802, center_y: 23.487, and center_z: 14.622 with dimensions size_x: 15.75, size_y: 15, and size_z: 15. The value of exhaustiveness was set to 20. The parameters energy_range and num_modes were set to 3 and 9, respectively. The best-scoring pose as judged by the Vina docking score was chosen and visually analyzed using PyMOL 1.7.6 software (http://www.pymol.org/ (accessed on 26 March 2017)). 

## 4. Conclusions

Screening dual site inhibitors of AChE is of great significance in AD drug research. In this paper, a natural bisesquiterpenoid, biatractylenolide II, was evaluated for its AChE inhibition activity in vitro. The results showed that biatractylenolide II had potential activity against AChE. The molecular docking study discovered that biatractylenolide II bound interactions with both CAS and PAS of the enzyme. In addition, the calculated log *p*-value also suggests that biatractylenolide II might pass easily through the blood–brain barrier. In conclusion, biatractylenolide II could be considered as a new lead compound to research and develop more potent dual site inhibitors of AChE. 

## Sample Availability

Samples of the compounds are not available from the authors.
